# SBRM-DB: Sugar beet root maggot database

**DOI:** 10.6026/9732063002001841

**Published:** 2024-12-31

**Authors:** Sudha Acharya, Nadim W Alkharouf, Muhammad Massub Tehseen, Chenggen Chu, Vincent P. Klink

**Affiliations:** 1 Department of Computer and Information Sciences, Towson University, Towson, MD, 21252, USA; 2 Department of Plant Sciences North Dakota State University Dept 7670, PO Box 6050 Fargo, ND 58108-6050 USA; 3 Department of Agriculture-ARS-Northern Crop Science Lab Edward T. Schafer Agricultural Research Center Fargo, ND 58102, USA; 4 Department of Agriculture-ARS-NEA-BARC Molecular Plant Pathology Laboratory Building 004, Room 122 BARC-West 10300 Baltimore Ave. Beltsville, MD 20705, USA

**Keywords:** Sugar beet root maggot, *Tetanops myopaeformis*, sugar beet, *Beta vulgaris*, genome, database

## Abstract

Sugar beet (SB), *Beta vulgaris* ssp, vulgaris (*B. vulgaris*), is one of only two plants in the world from which significant amounts of raw sugar is produced. This value of sugar, derived from SB, is 55% in the United States and 35% of global raw sugar with an annual farm worth in the U.S. alone of $1B, $4.6B globally. *Tetanops myopaeformis* (von Röder), the sugar beet root maggot (SBRM), is a devastating insect pathogen of SB and the most devastating SB pathogen in North America, decreasing production by up to 100%. The *T. myopaeformis* TmSBRM_v1.0 draft genome has been generated from DNA isolated from field-grown *B. vulgaris* from North Dakota, USA. A genome database for the annotated *T. myopaeformis* TmSBRM_v1.0 draft genome, SBRM database, has been generated and is presented here with the aim of aiding in agronomic improvement of SB for stakeholders.

## Background:

Sugar beet (SB), non-native to the U.S, is an important food crop in the US, with a $1.0B value, annually, harvested from 1.14 million acres of land and is one of only two plants, globally, from which sugar is widely produced with a worldwide value of $4.6B [1]. Apon SB's introduction to the U.S., it was encountered by the native *T. myopaeformis* (SBRM), capable of completing its life cycle on it through its pathogenic nature [2, 3]. Notably, the native SBRM host has not yet been identified, although the SBRM can complete its life cycle on other non-native plant species [4]. Of the North American SB diseases SBRM is the most devastating pathogen, decreasing yield by up to 100%, locally, with its spread increasing [3, 4, 5-6]. Annotated genomes for SB and SBRM are providing basic knowledge for the improvement of SB and food security [7]. The disease-causing habit of some species of the Tetanops genus indicates that the pathogenic life cycle may have aspects that are conserved between its species and are under genetic control so it may be possible to understand the pathogenic nature of SBRM in ways that would be facilitated by genomic information [8, 9, 10, 11, 12, 14-15]. The SBRM database that is presented here is aimed at providing a resource for the scientific community that can take the TmSBRM_v1.0 and future updated genome resources, RNA-seq and other omics data for employment in user-defined inquiries, including those targeted to interfere with the pathogenic nature of the SBRM with the work presented here describing the data and explaining its utility to the community while also providing a documented data link that is in a standard, re-useable format.

## Methodology:

## Construction of website database:

The sugar beet root maggot database (SBRM-DB, https://bioinformatics.towson.edu/SBRM) has been designed and implemented to manage the sequencing of *T. myopaeformis* genome, its annotations and related features, found at bioinformatics.towson.edu/sbrm/. By performing these actions, it allows users to implement designed queries and obtain information that may not, otherwise, be readily available or apparent.

The de novo assembly of the *T. myopaeformis* TmSBRM_v1.0 draft genome that was used to create the SBRM database has been uploaded to NCBI; Bio Sample accession: SAMN37733483, Bio Project ID PRJNA1026092, available at the URL: https://www.ncbi.nlm.nih.gov/bioproject/PRJNA1026092. The PacBio HiFi reads were assembled using the pipeline Flye, version 2. 9. 2. Default values were used, except for setting the --asm-coverage argument to 50, to reduce memory consumption. Flye was installed and run on the Windows Subsystem for Linux (Ubuntu 22.04), running on a Windows 2022 workstation with 45 GB of memory. The *T. myopaeformis* mate-pair library produced a total number of raw reads of 6,356,906. The total read length was 71,844,227,661 base pairs (bp) and the N50/N90 reads were 11,313 and 8,294, respectfully. The assembly statistics showed a total length of 414,327,873 bp with the number of contigs of 8,228. The contig's N50 was 57,402. The largest contig was 573,329 bp. The mean genome coverage was 94x. The SBRM database stores essential data relating to *T. myopaeformis* and retrieves the data based on gene identification (geneID) or other searchable parameters. The database stores descriptions of each gene, eukaryotic orthologous groups (KOG), gene ontology (GO) assignments and protein families (PFAM). The SBRM database has been designed implemented and hosted using Microsoft SQL Server 2016 Enterprise Edition. The SBRM database web application has been designed and implemented using ASP.NET with C# programming language which relies on the integrated development environment Microsoft Visual Studio 2017. The operating system that has been used for the server is Microsoft Windows Server 2012 and Internet Information Services version 7.0. The bioinformatics server at Towson University in Towson, MD, USA hosts the database and website of SBRM database. We have developed a user-friendly database-driven website that allows users to access all the stored data. Users can browse, search and download the data using gene IDs or descriptions. In addition, users can compare the differential gene expression results in the different samples.

## Utility and Discussion:

**Figure 1:**
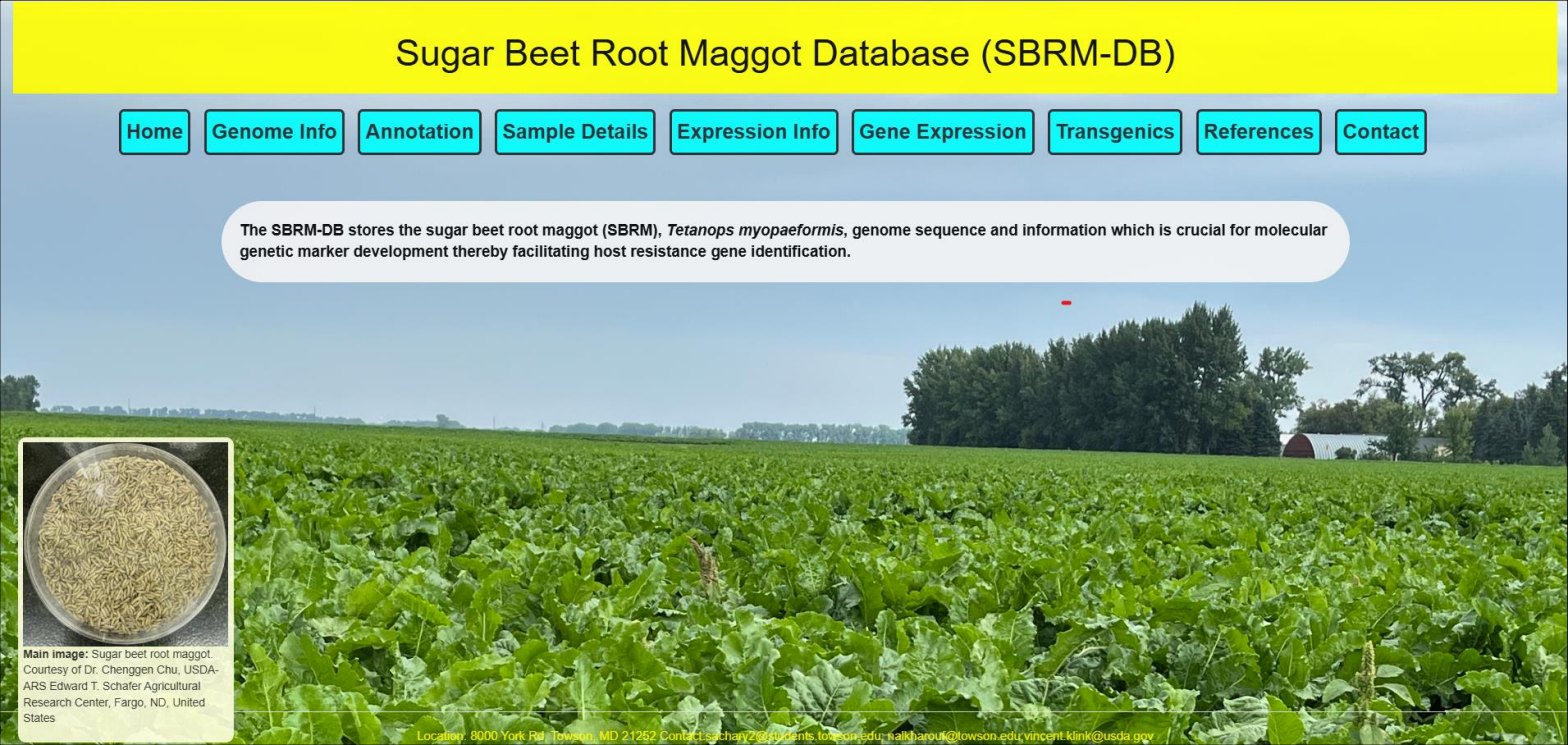
A snapshot of the SBRM database main web page.

An organism's genome encodes information used to accomplish its biological needs. The *T. myopaeformis* genome was sequenced, assembled and annotated with the goal of applying it to understanding the SBRM life cycle for stakeholder needs [9, 10]. The webpage is the user interface to user-defined queries (Figure 1). The nucleotide information of TmSBRM_v1.0 is foundational, important to map gene expression data obtained from the same population of SBRM from which the genome was generated. The TmSBRM_v1.0 provides crucial information in an insect family having great negative agricultural impact, serving as a research guidepost for agricultural management and genetic improvement of sugar beet. The annotation led to the identification of 28,276 genes with the gene annotations being categorized into biological process, cellular component, molecular function and transposable elements. Within the annotations the gene features include sequence name (SeqName), gene description (Description), length of the sequence (Length), #Hits, e-Value, sim mean, gene ontology (GO) number (#GO), GO identifier (GO IDs), GO Names, enzyme code (EC) (Enzyme Codes), names of the enzymes (Enzyme Names), InterPro IDs, InterPro GO IDs and InterPro GO Names.

## Browse:

It is possible to query any sequence that is present within this genome. Furthermore, the site is a database driven, searchable site. The site allows the user to retrieve the data freely for any needed use. Gene sequences that do not match to the TmSBRM_v1.0 genome are available as they may have important biological information [16].

## Search:

The SBRM database allows users to search the sequence information in two separate ways. Users can search sequence data through gene ID, or its accompanying description. When users search by gene ID, the exact gene ID must be entered in the text box to get the matched result. Alternatively, partial characters or text can be entered into searches for genes if the user searches by description. The different searches return their query results in a table that also shows the sequence data.

## Gene expression results:

Once anticipated gene expression results are imported, users can select a specific sample and retrieve a list of all the transcripts in that sample. Furthermore, the percentage of reads that align to the genome can be determined. Query outputs also provide their accompanying differential gene expression results (output from DEGSeq). The user can also narrow down the results by searching for specific gene(s) in the analysis. This is done by typing the exact gene ID(s) in the text box to get the gene information that matches those genes. In addition, the user can narrow down the results by searching for gene descriptions or parts of a description. This task is accomplished by typing the partial character(s) in the text box that exists beside the Search by Description identifier.

## Comparing all samples:

The SBRM database empowers users to compare any two samples. The search will compare the differential expression analysis results from each of the selected samples. In this web page, the web application enables users to retrieve the genes that are induced or suppressed in the first selected sample and induced or suppressed in the second sample. This search allows users to compare samples with their controls. All these queries return their results in a user-friendly table with the user being able to download the data to an excel file.

## Transgenics:

Other information in the database includes transgenics. This page will provide information and resources for the genetic engineering of sugar beet. The resource will aid in the translation of information like that gained from genetics and gene expression work to the improvement of sugar beet.

## Conclusion:

The sugar beet root maggot genome database, SBRM database, is presented. In addition to its home page, the SBRM database advances science by currently providing searchable links for genome information, gene annotations, experiment sample details, gene expression details, transgenic information, references and contact information. The goal for the development of the SBRM-DB is to make information available to the public for the improvement of sugar beet and related uses.

## Declaration of competing interests:

The authors declare that they have no known competing financial interests or personal relationships that could have appeared to influence the work reported in this paper.
